# Smoking and Early COPD as Independent Predictors of Body Composition, Exercise Capacity, and Health Status

**DOI:** 10.1371/journal.pone.0164290

**Published:** 2016-10-13

**Authors:** Laura Miranda de Oliveira Caram, Renata Ferrari, André Luís Bertani, Thaís Garcia, Carolina Bonfanti Mesquita, Caroline Knaut, Suzana Erico Tanni, Irma Godoy

**Affiliations:** Department of Internal Medicine, Pneumology Area, Botucatu Medical School, UNESP - Univ Estadual Paulista, Botucatu, São Paulo, Brazil; Lee Kong Chian School of Medicine, SINGAPORE

## Abstract

The effects of tobacco smoke, mild/moderate COPD disease and their combined effect on health status (HS), body composition (BC), and exercise capacity (EC) impairment are still unclear. We hypothesized that smoking and early COPD have a joint negative influence on these outcomes. We evaluated 32 smokers (smoking history >10 pack/years), 32 mild/moderate COPD (current smokers or former smokers), and 32 never smokers. All individuals underwent medical and smoking status evaluations, pre and post-bronchodilator spirometry, BC [fat-free mass (FFM) and FFM index (FFMI)], EC [six-minute walk distance (6MWD)] and HS [Medical Outcomes Study 36-Item Short-Form Health Survey (SF-36)]. FFM (p = 0.02) and FFMI (p = 0.008) were lower in COPD than never smokers. 6MWT, as a percentage of reference values for the Brazilian population, was lower in COPD and smokers than never smokers (p = 0.01). Smokers showed worse SF-36 score for functional capacity than never smokers (p<0.001). SF-36 score for physical functioning (p<0.001) and role-emotional (p<0.001) were impaired in COPD patients than smokers. SF-36 scores for physical functioning (p<0.001), role-physical (p = 0.01), bodily pain (p = 0.01), vitality (p = 0.04) and role-emotional (p<0.001) were lower in COPD than never smokers. Multiple linear regression analysis showed that both COPD diagnosis and smoking were inversely associated with FFMI, 6MWD and HS. Smoking and early COPD have a joint negative influence on body composition, exercise capacity and health status.

## Introduction

Smoking is a chronic disease and the leading preventable cause of death worldwide. It is related to more than 50 diseases, the most common being cancer in many sites, chronic obstructive pulmonary disease (COPD), and cardiovascular disease [1, 2]. COPD is characterized by persistent airflow limitation, which is usually progressive and associated with an enhanced chronic inflammatory response in the airways and lung to noxious particles or gases [3]. Furthermore, the disease imposes a high burden on healthcare systems worldwide and is currently the fourth leading cause of mortality [4].

Evidence suggests that smoking and the early stages of COPD have deleterious effects on skeletal muscles, exercise capacity, and health status (HS). The exposure to cigarette smoke *per se* can induce skeletal muscle dysfunction and smokers do suffer from greater peripheral muscle fatigue [5, 6]. Recent studies have not shown differences in body composition between smokers and never smokers; however, they have shown that smokers without airflow limitation had lower exercise capacity and worse SF-36 mental health dimension scores than nonsmokers [7, 8]. In addition, light-to-moderate smokers with a short smoking history, showed significant impairment in HS compared to never smokers [9].

Mild/moderate COPD may affect structural and functional abnormalities of skeletal muscles and reduce endurance even in the absence of significant muscle wasting [10–12]. Body composition in mild/moderate COPD is described as similar to healthy subjects, with decreased fat-free mass or higher fat percentage [13–15]. However, a decline in exercise capacity seems to occur in mild/moderate COPD [16] mainly in those with dyspnea when compared to smoker controls [17]. Few studies have compared HS in COPD patients in the early disease stages because it often goes undiagnosed and results are controversial [18–20].

As shown, the systemic effects of tobacco smoke and mild/moderate COPD disease are still unclear. Our hypothesis is that smoking and early COPD have a combined influence on systemic effects. Therefore, the aim of this study was to evaluate the influence of smoking and/or early COPD in skeletal muscle dysfunction, body composition, exercise capacity, and health status. Since oxidative stress and inflammatory mediators could interfere in the pathophysiology of these changes [21–23], we evaluated the influence of a general marker of oxidative stress (AGEs) and of systemic inflammation (TNF-alpha receptor 1) in the study variables.

## Material and Methods

From March 2013 to November 2014, 157 individuals (never smokers, smokers and patients with mild/moderate COPD) from the Pulmonology Outpatient and Smoking Cessation units at Botucatu Medical School were evaluated and 96 included in a cross-sectional study. Sixty-one patients were excluded according to exclusion criteria. Never smokers, active smokers (smoking history >10 pack / years) or mild/moderate COPD patients (smoker or former smoker, post-bronchodilator FEV_1_/FVC <0.70 and FEV_1_>50%) [3] were consecutively selected. Exclusion criteria included primary diagnosis of other respiratory diseases such as asthma, restrictive disorders (tuberculosis sequelae, interstitial fibrosis), sleep apnea/hypopnea syndrome, or lung cancer. In addition, a primary diagnosis of unstable angina, congestive heart failure (New York Heart Association class III or IV) or other chronic diseases, such as uncontrolled diabetes mellitus, kidney or liver failure, and cancer were also grounds for exclusion. Patients with mild/moderate obstruction were asymptomatic and not on maintenance medications. After initial screening, those included were evaluated over three days in the same week.

Sample size was calculated for a multiple linear regression effect size of 0.15 with an estimated multiple correlation squared of 0.50 with the addition of five predictors in the model (G Power 3.1.3).

Participants were made aware of the proposed study procedures and provided written informed consent. All procedures were approved by Botucatu Medical School University Hospital Research Ethics Committee (IRB approval number 410/08).

### Pulmonary function, pulse oximetry, smoking status, and dyspnea score

Pre and post-bronchodilator spirometry were performed using a KOKO spirometer (Ferrari KOKO Louisville, CO 80027, USA) according to criteria set by the American Thoracic Society (ATS) [24]. FEV_1_ values were expressed in liters and as percentages of FVC and reference values [25]. Pulse oximetry (SpO_2_) was assessed using an Onyx oximeter (Model 9500 Oximeter; Nonin Medical Inc.; Minneapolis, MN, USA) while the patients were breathing room air. Smoking history and current smoking state were investigated and complemented by assessing nicotine dependence intensity (Fargeström Test) [26]. Subjects were considered former smokers when abstinent for at least one year. Confirmation of smoking status was performed by measuring carbon monoxide (CO) in exhaled air by standardized technique (Micro CO Meter, Cardinal Health, England, UK). Value exhaled CO>6.0 ppm was considered active smoking [27,28]. Dyspnea was evaluated by Borg scale dyspnea score [29].

### Nutritional assessment

Body weight and height were measured by Filizola^®^ scale and body mass index ([BMI] = weight [kg]/height [m^2]^] calculated. Body composition was evaluated by bioelectrical impedance (BIA 101, RJL systems, Detroit, MI, USA) according to European Society for Parenteral and Enteral Nutrition guidelines [30]. Fat-free mass (FFM, kg) was calculated using a group-specific regression equation developed by Kyle et al. [30]. FFM index (FFMI = FFM/height^2^) was also calculated. Lean body mass depletion was defined as an FFMI<15 kg/m^2^ for women and <16 kg/m^2^ for men [31].

### Exercise capacity

The six-minute walk distance (6MWD) was performed according to ATS guidelines [32]. Before and after the test, data were obtained for SpO_2_, heart rate, respiratory rate, Borg scale dyspnea score [29], and blood pressure. The distance covered was reported in meters and as percentage of predicted distance according to the equation proposed by Iwama et al. for the Brazilian population [33].

### Peripheral muscle strength (handgrip and lower limb strength)

Handgrip strength was assessed by a manual dynamometer (SAEHAN Corp. Masan, Korea). Generated power was read in kilograms-force (kgf) and the highest of three readings was used for data analysis [34]. Lower limb muscle strength was performed using a portable MicroFet 2 dynamometer (measured in pounds and later converted to Newtons) (Hoggan Health, USA) [35, 36]. Participants performed up to seven trials, with a 10s rest between trials; the highest of three reproducible measurements within 5% of one another was used for analysis as described by Coronell et al. (2004) [37, 38]. The same examiner performed both evaluations.

### Advanced glycation end products (AGEs) and Tumor necrosis factor alpha (TNF-alpha) receptor 1 (TNFR1)

Fasting peripheral blood was collected and plasma stored at -80°C until analysis. AGEs and TNFR1 were measured in plasma by ELISA (Cell Biolabs, Inc. San Diego, CA, USA). The readings were obtained in a Spectra Max 190 microplate spectrophotometer (Molecular Devices^®^, Sunnyvale, CA, USA).

### Health status

A translation, validated for use in Brazil, of the Outcomes Study 36-Item Short-Form Health Survey (SF-36) was used to evaluate patient health status [39].

### Statistical analysis

Descriptive statistics were used to describe the features of all participants. Means ± SD or medians and interquartile range (25–75%) were used depending on data distribution. Categorical variables were expressed as percentages. Chi-square was used to compare the values of categorical variables.

Analysis of variance (ANOVA) followed by the Tukey or Kruskal-Wallis test followed by Dunn's test was used to compare demographic and general characteristics (e.g.: spirometry, lean body mass, peripheral muscle strength, and exercise capacity) between smokers, mild/moderate COPD, and never smokers.

Multiple linear regression analysis was used to assess associations between COPD (absence = 0 and presence = 1), smoking status (absence = 0 and presence = 1), exercise capacity, health status, body composition, systemic inflammatory state, and oxidative stress in the three study groups. Setting variables in the model were: gender and age. Collinearity was prevented by deleting a variable showing correlation. The level of significance was set at 5%. All analyses were performing using IBM SPSS Statistics 22 and Sigma Plot 11.0.

## Results

Table 1 shows the characteristics of the 96 subjects enrolled in the study according to the following groups: never smokers, smokers and mild/moderate COPD. COPD patients were older and presented lower body composition values than never smokers. When compared to former smokers COPD, current smokers COPD showed lower FFMI [Current smokers COPD: 17.7 (16.2–18.5) kg/m^2^ vs Former smokers COPD: 21.8 (17.2–26.9) kg/m^2^, p = 0.01]. Smokers and COPD showed lower spirometric values and higher AGEs concentrations than never smokers. Systemic inflammation, evaluated by TNFR1, was not different between groups (Table 1).

**Table 1:** General characteristics of individuals in the three study groups.

Variables	Never smokers (n = 32)	Smokers (n = 32)	COPD I/II (n = 32)	p Value
Age (years)	49.5 (46.0–58.5)a	53.0 (51.0–55.0)ab	64.5 (58.0–74.5)b	**<0.001**
Sex (M/F)	9/23	7/25	12/20	0.75
Weight (kg)	70.0 (60.6–84.5) a	68.5 (58.5–74.7) ab	60.0(49.5–71.2) b	**0.01**
Height (m)	1.63 ± 0.07	1.61 ± 0.08	1.59 ± 0.09	0.07
BMI (kg/m2)	26.8 ± 4.5	25.8 ± 3.6	24.4 ± 4.2	0.07
FFM (kg)	47.6 ± 8.4a	46.8 ± 8.9ab	41.5 ± 10.7b	**0.02**
FFMI (kg/m2)	23.3 (20.3–29.8)a	19.8 (17.4–24.3)ab	18.4 (16.7–22.5)b	**0.008**
Current smokers (n/%)	0/0	32/100	14/44	**<0.001**
Smoking history (pack-years)	0a	38.0 (21.5–50.7)b	55.5 (39.5–77.5)c	**<0.001**
CO (ppm)	0a	10.0 (8.2–15.7)b	0.0 (0–12.0)c	**<0.001**
FVC (L)	3.6 (3.1–4.0)a	3.0 (2.6–3.4)bc	2.7 (2.3–3.4)c	**<0.001**
FVC (% predict)	102.0 (93.0–110.5)	97.0 (90.0–103.0)	93.0 (79.5–103.0)	**0.06**
FEV1 (L)	2.9 ± 0.4a	2.3 ± 0.5b	1.8 ± 0.5c	**<0.001**
FEV1 (% predict)	100.8 ± 12.9a	91.1 ± 14.5b	77.4 ± 17.0c	**<0.001**
FEV1/FVC	0.81 (0.79–0.85)a	0.78 (0.73–0.83)b	0.63 (0.59–0.67)c	**<0.001**
SpO2 (%)	96.0 (95.0–97.5)	96.0 (95.0–97.0)	95.0 (93.0–97.0)	0.16
Borg	0 (0–0)	0 (0–0)	0 (0–0)	1.00
AGEs (μg/mL)	0.003 (0.002–0.004)a	0.005 (0.004–0.006)b	0.005 (0.004–0.006)bc	**<0.001**
TNFR1 (mg/L)	119.2 (104.3–139.5)	122.8 (103.7–136.7)	134.0 (115.7–210.3)	0.07

Values expressed as mean ± standard deviation or median (quartile 1—quartile 3). COPD I/II: mild/moderate (GOLD: Global Initiative for Chronic Obstructive Lung Disease); kg: kilograms; m: meters; BMI: body mass index; FFM: fat-free mass; FFMI: fat-free mass index; CO: carbon monoxide; FEV_1_: forced expiratory volume in one second; FVC: forced vital capacity; SpO_2_: pulse oximetry; AGEs: advanced glycation final products; TNFR1: tumor necrosis factor alpha receptor 1. a, b, c: Different letters indicate statistically significant difference. p <0.05. χ 2, ANOVA and Tukey or Kruskal-Wallis test and Dunn's test.

### Peripheral Muscle Strength and Exercise Capacity

**Fig 1:**
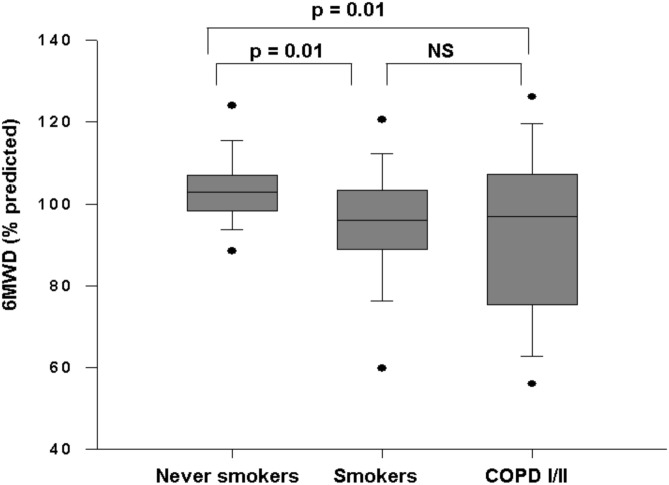
Percentage values of predicted exercise capacity evaluated by 6MWD p<0.05 (Kruskal-Wallis and Dunn's test).

The peripheral muscle strength did not differ between groups. Average 6MWD in the control group was significantly higher than COPD patients [COPD patients: 492.0 (390.0–562.0) m vs Smokers: 515.5m (477.0–570.0m) vs Never smokers: 554.5m (516.5–606.0m), p = 0.004]. When calculated as a percentage of predicted values by Iwama et al. COPD patients and smokers presented lower values than never smokers (Fig 1). Predicted values did not differ between smokers and mild/moderate COPD.

### Health Status

**Fig 2:**
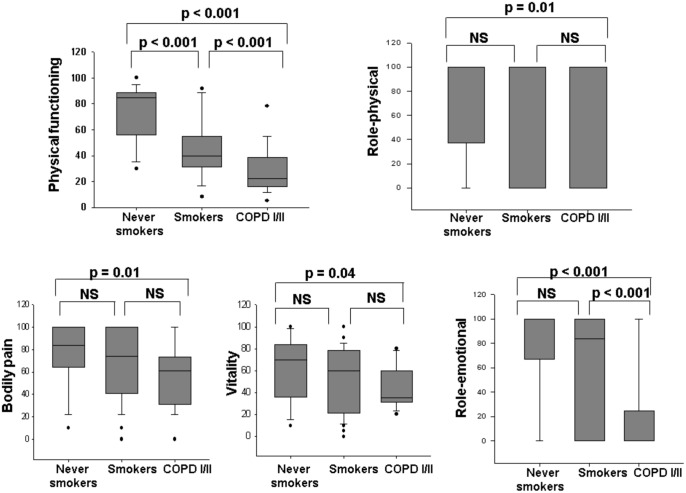
Health Status evaluated by SF-36; p<0.05 (Kruskal-Wallis and Dunn's test).

The smokers group showed worse SF-36 scores for functional capacity than never smokers and better scores for SF-36 physical functioning and role-emotional domains than COPD (Fig 2). Patients with COPD showed significantly impaired health status than never smokers in the following SF-36 domains: physical functioning, role-physical, bodily pain, vitality and role-emotional (Fig 2). There were no statistically significant differences in the others domains (Table 2).

**Table 2:** Health Status evaluated by SF-36 of the individuals according studied groups.

*Variables*	*Never smokers (n = 32)*	*Smokers (n = 32)*	*COPD I/II (n = 32)*	*p-value*
*SF-36*				
General health	74.5 (52.0–82.0)	57.0 (47.5–82.0)	42.0 (32.0–77.0)	0.08
Social functioning	69.0 (50.0–100.0)	75.0 (44.0–100.0)	63.0 (25.0–75.0)	0.05
Mental health	58.0 (16.0–88.0)	66.0 (34.0–84.0)	32.0 (28.0–78.0)	0.43

Values expressed as median (quartile 1—quartile 3). COPD I/II: mild/moderate (GOLD: Global Initiative for Chronic Obstructive Lung Disease. SF-36 Medical Outcomes Study 36-item Short-Form Health Survey. ANOVA and Tukey or Kruskal-Wallis test and Dunn's test.

Multiple linear regression analysis identified smoking and the presence of COPD as independent predictors of body composition (FFMI), 6MWD, and health status (SF-36 physical functioning score) (Table 3). In other models, we analyzed the association of the smoking history (packs-year) and of the interaction smoking x COPD in the dependent variables (FEV_1_/FVC, FMMI, 6MWD, and Physical functioning) and no association found (data not shown).

**Table 3:** Predictors of body composition (FFMI), exercise capacity (6MWD) and health status (SF-36 physical functioning score).

*Dependent Variable*	*Variables*	*Coefficient (95% IC)*	p value
**FFMI (R**^**2**^ **= 0.57)**	Age, years	-0.17 (-0.20; 0.0)	0.06
	Gender, male	0.67 (6.97; 10.89)	<0.001
	Smoking (presence)	-0.21 (-4.38; -0.84)	**0.004**
	COPD (presence)	-0.23 (-5.13; -0.80)	**0.008**
	TNFR1, mg/L	0.11 (-0.00; 0.03)	0.15
**6MWD (R**^**2**^ **= 0.20)**	Age, years	-0.12 (-3.08; 1.16)	0.37
	Gender, male	0.23 (5.91; 84.54)	0.02
	Smoking (presence)	-0.21 (-73.80; -2.01)	**0.03**
	COPD (presence)	-0.25 (-93.07; -2.76)	**0.03**
	TNFR1, mg/L	-0.12 (-0.56; 0.16)	0.27
**Physical functioning (R**^**2**^ **= 0.32)**	Age, years	-0.13 (-1.13; 0.26)	0.22
	Sex, male	-0.06 (-19.12; 8.85)	0.46
	Smoking (presence)	-0.32 (-35.02; -9.04)	**0.001**
	COPD (presence)	-0.35 (-41.16; -10.28)	**0.001**
	AGEs, μmg/mL	-0.15 (-5232.99; 338.51)	0.08

COPD: chronic obstructive pulmonary disease; FFMI: fat-free mass index; TNFR1: tumor necrosis factor alpha receptor 1; 6MWD: six-minute walk distance; AGEs: advanced glycation final products.

## Discussion

The main results of this study showed that active smoking and early COPD have a cumulative negative influence on lean body mass, exercise capacity, and health status. To our knowledge, this cumulative effect has not been shown previously and supports the importance of therapeutic measures to promote smoking cessation, physical activity and prevent the progression of airflow obstruction in smokers and COPD patients even in the absence of symptoms.

Early COPD showed a clear FFM compromise compared to never smokers and we found a negative association between smoking and COPD presence with FFMI. Studies evaluating FFM including smokers and mild/moderate COPD are scarce and controversial [13–15]. Our data support recent findings showing that smoking influences a reduction in lean mass [40] and that smokers have lower appendicular skeletal muscle mass index than nonsmokers [41]. A positive association between FFM and spirometry variables (FEV_1_, FVC and FEV_1_/FVC), and reduced FFM in ex-smokers with mild COPD compared to healthy subjects have also been reported [13,42]. However, Puig-Vilanova et al. did not find any difference in body composition (BMI and FMMI) between mild COPD and controls [15]. In addition, Rutten et al. (ECLIPSE) in a follow-up study found that changes in body composition over time were similar in COPD as compared to controls (both smoking and nonsmoking). As no follow-up of our sample was made, we cannot comment in differences in body composition in smokers and early COPD overtime [43].

Smoking *per se* and COPD diagnosis seem to have cumulative effects on body composition and there are data to reinforce this finding. Tobacco use could lead to weight loss by increasing the metabolic rate, decreasing metabolic efficiency or caloric absorption (appetite reduction) [44]. A reduction in Type I and an increase in Type IIb fibers, as well as reduced muscle oxidative capacity were shown in smokers [45]; similar findings have been reported in COPD patients [16,46]. There is a growing appreciation that structural and functional abnormalities in skeletal muscles (muscle injury, loss of skeletal muscle oxidative capacity and diminished endurance) can be present in patients with mild/moderate COPD and may develop in the absence of significant muscle wasting [12,47]. Triggers for muscle wasting in early COPD include disuse, inflammation, and sarcopenia. Muscle wasting (sarcopenia) is systemic in many diseases, including COPD. Rapid loss of muscle mass and strength primarily results from excessive protein breakdown which is often accompanied by reduced protein synthesis [48]. A sedentary lifestyle is a common characteristic of COPD and affects peripheral skeletal muscle mass and function; and even in mild COPD, physical inactivity is associated with quadriceps wasting [49]. Moreover, elevated circulating levels of inflammatory markers and their soluble receptors in COPD patients have been associated with acute weight loss and reduced lean mass [49].

Importantly, muscle wasting not only contributes to diminished muscle function, but also reduces exercise capacity [49]. Few studies have compared exercise capacity in smokers and early COPD patients and results are controversial [7,8,16,17]. Although mean 6MWT (m) was not different between smokers and never smokers, the 6MWD (% predicted) was lower in smokers than never smokers, which agrees with recent findings [8]. However, Furlanetto et al. found a decrease in mean 6MWT (m) in the smoker group compared to non-smokers [7]. In mild/moderate COPD, Eliason et al. observed a 21% decrease in distance walked compared to age-matched healthy subjects [16]. Díaz et al. showed a more pronounced decrease in walking distance in mild dyspneic COPD compared to non-dyspneic mild COPD and smoker controls [17]. The possible factors related to the deleterious effects of smoking on exercise capacity are peripheral muscle abnormalities and deterioration in body composition [16,44–46,48]. In addition, perturbation in muscle energy metabolism may also contribute to exercise intolerance in COPD [47] as direct measurements of muscle metabolites in biopsy samples showed muscle alterations in COPD patients [50].

Our patients were asymptomatic and even with the absence of a difference in peripheral muscle strength there was a difference in exercise capacity. In fact, 6MWD reflects daily activities and is used to evaluate the integrated response of the pulmonary, cardiovascular, and muscular systems [32]. Increased blood CO can reduce exercise tolerance and maximal aerobic capacity [51] and even in mild COPD, aerobic capacity (evaluated by progressive cycle ergometry) decreased and end-expiratory lung volume increased leading to dynamic hyperinflation and a limitation in ventilatory response to exercise [52].

In this study, smokers and early COPD patients had impaired health status compared to never smokers. Previous data indicate the negative effects of smoking on health status [9,20,53]. According to Martinez et al. even light-to-moderate smokers with a short smoking history show a significant deterioration in health status [9] and a study evaluating changes in health status twelve months after smoking cessation showed a significant improvement in 40 ex-smokers compared to 20 active smokers [53]. Mild/moderate COPD patients showed a lower physical score than non-COPD individuals [19] and a negative association was seen between health status and presence of COPD [20]. Similarly, a recent study has suggested health status is impaired even in patients with the mild form of the disease [18]. However, Janson et al. showed that only moderate COPD patients had impaired health status compared to non-COPD individuals. Health status did not differ between mild COPD and non-COPD subjects [20].

Hypothetical explanations for the decline in health status may initially be sought in cigarette consumption itself. Smoking produces thousands of chemicals that are absorbed through the lungs. Some substances could have organic actions leading to asthenia, vitality loss, muscle disorders, or psychological derangement [9]. Furthermore, the present study and additional investigations have shown increased AGE levels in smokers and a positive association between this marker and packyears [22,54]. Both smoking and oxidative stress shorten life and impair its quality [55]. According to our data, studies showed that smokers present psychological derangement. A recent study showed deterioration in the SF-36 questionnaire mental health domain [8] and an association between anxiety and depression scores and the presence of smoking has also previously been shown [56,57].

## Conclusions

Smokers and patients with early COPD have reduced lean body mass, and impaired exercise capacity and health status. Furthermore, smoking and early COPD have a negative influence on body composition, exercise capacity, and health status, resulting in cumulative negative effects. These findings reinforce the importance of therapeutic measures to promote smoking cessation and to prevent the progression of airflow obstruction.
